# Agronomic Approach of Zinc Biofortification Can Increase Zinc Bioavailability in Wheat Flour and thereby Reduce Zinc Deficiency in Humans

**DOI:** 10.3390/nu9050465

**Published:** 2017-05-06

**Authors:** Dunyi Liu, Yumin Liu, Wei Zhang, Xinping Chen, Chunqin Zou

**Affiliations:** Key Laboratory of Plant-Soil Interactions, Ministry of Education, Center for Resources, Environment and Food Security, China Agricultural University, Beijing 100193, China; jia57@126.com (D.L.); lymn@cau.edu.cn (Y.L.); zw0730@163.com (W.Z.); chenxp@cau.edu.cn (X.C.)

**Keywords:** zinc biofortification, wheat flour, phytic acid, zinc bioavailability, DALYs

## Abstract

Zinc (Zn) deficiency is a common disorder of humans in developing countries. The effect of Zn biofortification (via application of six rates of Zn fertilizer to soil) on Zn bioavailability in wheat grain and flour and its impacts on human health was evaluated. Zn bioavailability was estimated with a trivariate model that included Zn homeostasis in the human intestine. As the rate of Zn fertilization increased, the Zn concentration increased in all flour fractions, but the percentages of Zn in standard flour (25%) and bran (75%) relative to total grain Zn were constant. Phytic acid (PA) concentrations in grain and flours were unaffected by Zn biofortification. Zn bioavailability and the health impact, as indicated by disability-adjusted life years (DALYs) saved, increased with the Zn application rate and were greater in standard and refined flour than in whole grain and coarse flour. The biofortified standard and refined flour obtained with application of 50 kg/ha ZnSO_4_·7H_2_O met the health requirement (3 mg of Zn obtained from 300 g of wheat flour) and reduced DALYs by >20%. Although Zn biofortification increased Zn bioavailability in standard and refined flour, it did not reduce the bioavailability of iron, manganese, or copper in wheat flour.

## 1. Introduction

Zinc (Zn) is one of the most abundant trace elements in human bodies, with 1.5–2.5 g present in the average adult [1]. As a catalytic and an important structural component in an estimated 3000 zinc proteins, Zn is essential for carbohydrate metabolism, DNA and RNA synthesis, and other processes [2,3]. Zinc deficiency, however, is prevalent in many parts of the world and especially in developing countries [4]. Zinc deficiency results in retarded growth, anorexia, and hypogeusia in children [5], and in pregnancy problems and several chronic diseases in adults [6,7].

In more than 22 developing countries, at least 60% of the Zn in human diets is derived from C3 grains and legumes [8]. Wheat is one of the three leading cereal crops worldwide and is the dominant crop used for human food [9]. Worldwide wheat production exceeds 720 million tons per year, and most of which is used as food for humans [9,10]. Wheat can be processed into a rich variety of flours depending on the milling procedure. Whole grain flour and coarse flour are valuable sources of dietary fiber, and standard flour and refined flour are commonly used to make bread and noodles. Wheat has a low Zn concentration, with only about 20–35 mg/kg of whole grain [4]. The low concentration of Zn in wheat results in part from the low Zn content of soils where wheat is grown, i.e., more than 40% of the worldwide wheat crop is cultivated on soils with very low levels of Zn [11]. Furthermore, a substantial percentage of the Zn in grain is lost with the removal of the aleurone layer and embryo during milling [12]. Zinc concentrations were reported to be less than 15 mg/kg in wheat endosperm and in refined flour [13].

An important approach to preventing Zn deficiency in humans is Zn biofortification, i.e., the use of agronomic practices, conventional plant breeding, or biotechnology to increase the Zn content of food crops [4]. Rosado et al. [14], for example, reported that Zn intake was substantially higher for humans who consumed biofortified wheat than non-biofortified control wheat. Qin et al. [15] indicated that Zn-biofortified rice increases dietary Zn intake and reduces the risk of Zn deficiency. At least in the short term, Zn biofortification can be achieved more rapidly by an agronomic approach (i.e., by fertilizer application) than by conventional breeding or biotechnology [4].

In addition to being inherently low in Zn, wheat is rich in phytic acid (PA), a compound that limits Zn bioavailability [16]. Phytic acid is a powerful chelator that bind metals into insoluble compounds [17]. Fredlund et al. [18] concluded that the inhibitory effect of PA on the absorption of Zn was dose dependent, and that only 50 mg of phytate-P (269 mmol phytate) could significantly decrease the absorption of Zn in human meals. The PA:Zn molar ratio has been widely used as a simplified measure of Zn bioavailability in the human diet [19]. The human body regulates Zn homeostasis through gastrointestinal secretion and excretion of endogenous Zn in addition to absorption of exogenous Zn [20]. A trivariate model that considers Zn homeostasis in the human intestine has been widely used to evaluate the effects of Zn bioavailability in humans [21].

The burden of disease and injury in human populations is usually quantified in terms of disability-adjusted life years (DALYs) [22]. DALYs have been used to assess the burden of Zn deficiency and the benefits of Zn-biofortified wheat [23,24]. In calculating DALYs, however, researchers have sometimes used roughly estimated values for certain parameters. For example, the percentage of Zn retained in flour after milling is sometimes assumed to be 60% [23], but this percentage differs greatly depending on the kind of flour. Also, the Zn bioavailability in humans has previously been ignored in the calculation of the daily Zn intake [23,24]. In fact, the health impact of biofortified flour can be reliably assessed while taking Zn bioavailability into consideration.

Micronutrients such as iron (Fe), manganese (Mn), and copper (Cu) are also essential for wheat growth and human health. Research on the relationship between Zn and other micronutrients in wheat and in humans has produced inconsistent results. Majid et al. [25], for example, reported that Zn application to wheat generally increased the concentrations of Zn, Fe, and Mn in grain, while Zhao et al. [26] indicated that Zn application to wheat reduced Fe concentrations but did not affect Cu and Mn concentrations in grain. Kabata-Pendias and Pendias [27] reported that the Zn–Cu interaction was antagonistic. These discrepancies in results might be due to differences in environmental factors or plant genotypes. Such discrepancies have also been reported in humans [20]. For example, some studies have indicated that levels of serum or plasma ferritin decreased with Zn supply [28]; other studies found that these levels increased with Zn supply [29], and a meta-analysis indicated that Zn supply did not affect levels of serum or plasma ferritin [30]. The effects of Zn application in the field on the bioavailability of Fe, Cu, and Mn in wheat flour fractions have not been previously reported.

The objectives of this study were (1) to quantify the effects of Zn biofortification via fertilizer application on Zn and PA concentrations in wheat grain and in different kinds of flours; (2) to estimate the resulting bioavailability of Zn in humans; and (3) to assess the health impact of Zn-biofortified flours. The relationship between Zn biofortification and the bioavailability of other micronutrients was also evaluated.

## 2. Materials and Methods

### 2.1. Wheat Grain Biofortification

A field experiment was conducted in 2013–2014 and 2014–2015 at the Quzhou Experiment Station (36.9° N, 115.0° E) in China. In both cropping seasons, wheat was grown from October to the following June in a winter wheat–summer maize rotation system. The soil pH (1:2.5 *w*/*v* in water) was about 8.0, and the diethylene triamine pentaacetic acid (DTPA)-extractable Zn, Fe, Cu, and Mn concentrations before sowing were 0.45, 5.52, 0.82, and 5.30 mg/kg, respectively. The same winter wheat (*Triticum aestivum* L.) cultivar (Liangxing 99, Dezhou, China) was used in both cropping seasons. Six Zn application rates were assessed: 0, 10, 25, 50, 100, and 150 ZnSO_4_·7H_2_O (22% Zn) kg/ha. The experiment had a randomized complete block design with four block and 24 plots; each plot was 75 m^2^. Before sowing, a compound fertilizer (N-P_2_O_5_-K_2_O: 15-15-15; 75 kg/ha) was applied. Another 150 kg of N/ha was supplied as urea at the stem elongation stage. The plots were irrigated at the pre-wintering, stem elongation, and flowering stages in both cropping seasons. At maturity, wheat plants aboveground were removed by hand from a 6-m^2^ (2 m × 3 m) area in each plot. About 1 kg of grain from these plants was sampled and rapidly washed with deionized water before milling and analysis.

### 2.2. Flour Fractions and Analysis

Wheat samples were milled in a Bühler laboratory experimental mill (MLU-202) according to Approved Method 26-21A [31]. Three break streams (B1, B2, and B3), three reduction streams (R1, R2, and R3), and two brans (coarse bran and fine bran) were obtained after milling. Different percentages of break, reduction, and bran fractions were combined to make the different kinds of flour (Table 1). In this report, “whole flour” was the flour that contained all milling fractions (100% of the grain). “Coarse flour” contained the coarse bran fraction and included 86.4% of the grain. “Standard flour” represented the ordinary flour sold in markets; it was a mixture of B1, R1, B2, R2, B3, and R3 fractions and included 75.7% of the grain. “Refined flour” refers to the highly refined and purified endosperm and is usually used to make Chinese fine noodles; refined flour was a mixture of B1 and R1 fractions and included 47.1% of the grain. The term “bran” refers to the combination of coarse bran and fine bran and is usually used to feed animals. All stream samples were digested with HNO_3_-H_2_O_2_ in a microwave-accelerated reaction system (CEM, Matthews, NC, USA), and the nutrients in the digested solution were determined by inductively coupled plasma optical emission spectroscopy (ICP-OES, OPTIMA 7300 DV, Perkin-Elmer, Houston, TX, USA). PA concentrations in different fractions were determined calorimetrically (at 519 nm) as described by Haug andLantzsch [32].

### 2.3. Estimation of Zn Bioavailability

A trivariate model based on Zn homeostasis in the human intestine was used to evaluate Zn bioavailability [21]:

(1) $$TAZ=0.5\times 65\times 100\times \left\{A_{MAX}+TDZ+K_{R}\times \left(1+\frac{TDP}{K_{P}}\right)-\sqrt{{\left(A_{MAX}+TDZ+K_{R}\times \left(1+\frac{TDP}{K_{P}}\right)\right)}^{2}-4\times A_{MAX}\times TDZ}\right\}$$

where *TAZ* = total daily absorbed Zn (mg Zn/day); *A_MAX_* = maximum Zn absorption; *TDZ* = total daily dietary Zn (mmol Zn/day); *K_R_* = equilibrium dissociation constant of the Zn-receptor binding reaction; *TDP* = total daily dietary PA (mmol PA/day); and *K_P_* = equilibrium dissociation constant of the Zn–PA binding reaction. According to Hambidge et al. [33], the parameters related to Zn homeostasis in the human intestine, A_MAX_, K_R_, and K_P_, have constant values of 0.091, 0.680, and 0.033, respectively. *TAZ* was based on reference adults consuming wheat flour (300 g/day) as a sole daily source of Zn and phytate [14] and was termed “estimated Zn bioavailability”. Molar concentrations of phytate and Fe, Mn, and Cu in wheat grain were used to calculate the PA:Fe, PA:Mn, and PA:Cu molar ratios, which in turn were used to estimate the bioavailability of Fe, Mn, and Cu in wheat grain [34].

### 2.4. Health Impact of Zn Biofortification in Wheat

The DALYs equation was used to estimate the health burden of Zn deficiency and to assess the health impact of Zn-biofortified flours. The current burdens due to Zn deficiency were calculated based on a recent study in China, which indicated that years of life lost (YLL) was 151 million and years lived with disability (YLD) was 202 million years, resulting in a total DALYs value of 352 million years [24]. The status quo of daily Zn intake was 4.90 mg/day for infants and 6.00 mg/day for children [35]. The recommended nutrition intake (RNI) levels for Zn in developing countries was 6.90 mg/day for infants and 8.00 mg/day for children [36]. Relative to the reference daily flour consumption of 300 g/day, consumption by infants is about 25% (75 g/day) and consumption by children is about 50% (150 g/day). Daily Zn intake through biofortified flours was the current daily Zn intake (4.90 and 6.00 mg/day) plus the increased TAZ calculated by the above trivariate model. The “coverage rate” of biofortified wheat and flour (i.e., the percentage of the population that consumed biofortified wheat and flour) was set at 60%, which represents an optimistic scenario. We then calculated the health impact (DALYs saved) of the biofortified flour by the method ofStein et al. [37] and De Steur et al. [24]. We also calculated the health impact of Zn biofortification as follows: Health impact = health burden saved by Zn biofortification/health burden without biofortification × 100. In this equation, health burden is equivalent to DALYs.

### 2.5. Statistical Analysis

One-way ANOVAs were used for many comparisons; means were separated by Fisher’s protected least significance difference (LSD) test at *p* < 0.05. Statistical Analysis System software (SAS 8.0, Raleigh, NC, USA) was used for the statistical analysis.

## 3. Results

### 3.1. Zn and PA Concentrations in Grain Milling Fractions

**Figure 1:**
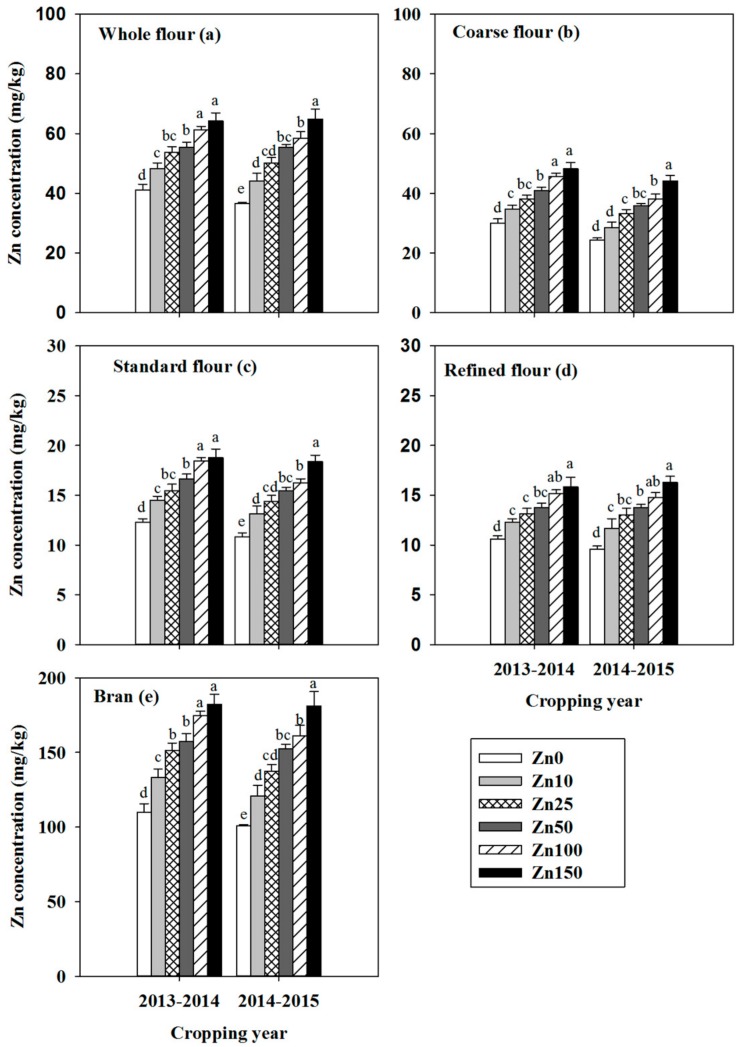
Zinc concentration in wheat whole flour (**a**); coarse flour (**b**); standard flour (**c**); refined flour (**d**); and bran (**e**) as affected by the quantity of Zn fertilizer applied (0 to 150 kg of ZnSO_4_·7H_2_O/ha) in 2013–2014 and 2014–2015. Values are means + SE (*n* = 4). Within each panel and cropping year, means with different letters are significantly different at *p* < 0.05. Note that the Y-axis scale is not constant among panels.

**Figure 2:**
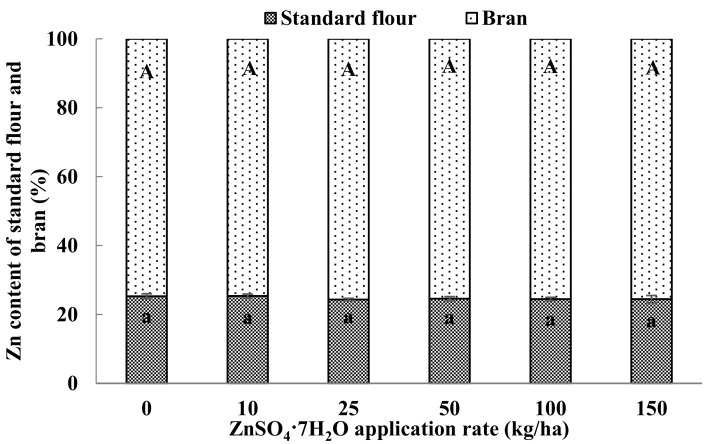
Zn content in standard flour and bran expressed as a percentage of the total Zn content in grain and as affected by the rate of Zn fertilization. Values are means (+SE) of two cropping years. Same letters in lower-case (a) and capital letters (A) means neither the percentages in standard flour nor in bran were significantly affected by the rate of Zn fertilization (*p* > 0.05).

In both cropping years, Zn concentrations in grain milling fractions were significantly greater in the Zn-biofortified treatments than in the no-Zn treatment, and the increases in Zn concentrations were closely related to the quantity of Zn applied (Table 2). The Zn concentrations were much greater in coarse bran and fine bran than in the break and reduction flour, and were 8–10 fold higher in bran than in standard flour. Zn concentrations in whole flour, coarse flour, standard flour, and refined flour increased with the rate of Zn fertilization. Among the four kinds of flour, the Zn concentrations were generally higher in whole flour and coarse flour than in standard flour and refined flour (Figure 1). Regardless of the quantity of Zn applied, the Zn content in bran accounted for 75% of the whole grain Zn while in standard flour it accounted for 25% (Figure 2). The PA concentration in the flours and bran was generally not significantly affected by Zn biofortification. The PA concentration was also much higher in the bran than in the other flours (Table 2).

### 3.2. Estimated Zn Bioavailability in Wheat Flours

**Figure 3:**
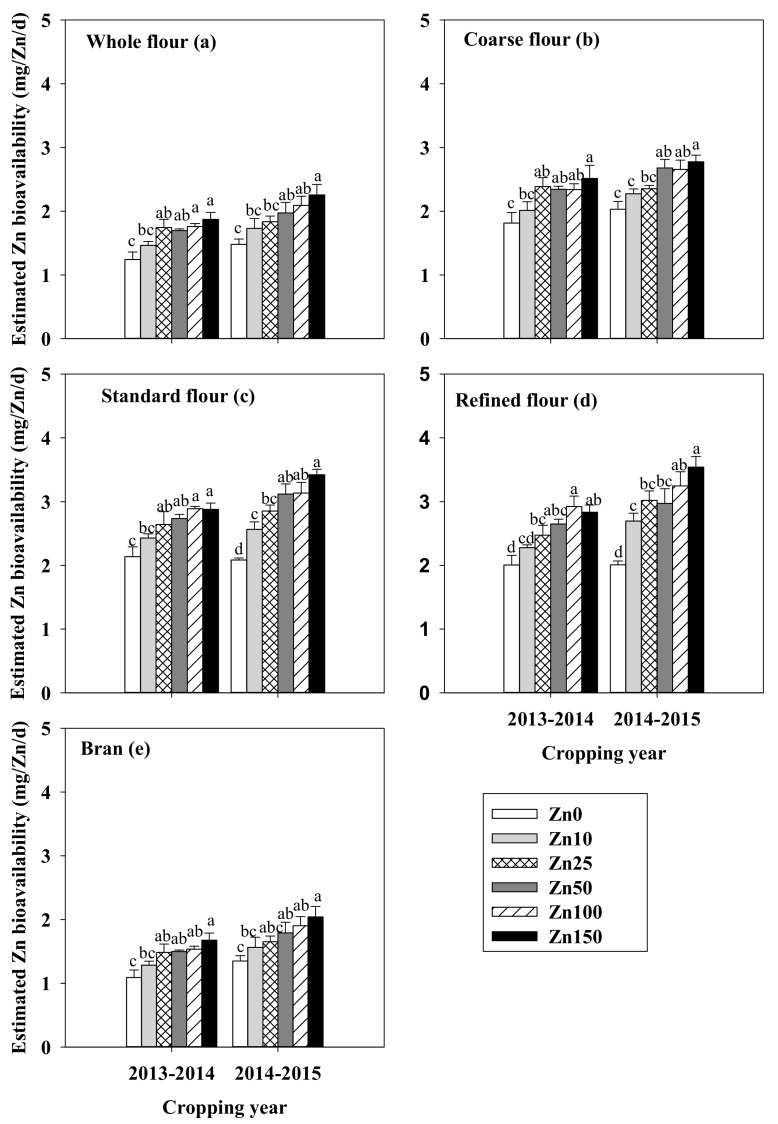
Estimated bioavailability of the Zn in whole flour (**a**); coarse flour (**b**); standard flour (**c**); refined flour (**d**); and bran (**e**) as affected by the rate of Zn fertilization (0 to 150 kg of ZnSO_4_·7H_2_O/ha) in 2013–2014 and 2014–2015. Values are means + SE (*n* = 4). Within each panel and cropping year, means with different letters are significantly different at *p* < 0.05.

The estimated Zn bioavailability in whole flour, coarse flour, standard flour, refined flour, and bran increased with the quantity of Zn applied in both cropping seasons (Figure 3). The estimated Zn bioavailability was generally higher in standard flour and refined flour than in whole flour and coarse flour (Figure 3).

### 3.3. Health Impact of Zn Biofortified Flours in China

In all four of the most commonly used flours (whole, coarse, standard, and refined), the daily Zn intake, the percentage of the recommended intake, and the DALYs saved for both infants and children were progressively increased as the rate of Zn fertilization increased (Table 3). The reductions in the current health burden of consuming biofortified whole flour and coarse flour were almost the same, ranging from 6.17% to 18.66%. Among the four flours, the health impacts (DALYs saved) of Zn biofortification of standard and refined flour were better than that of the other two flours. The estimated reductions in the current health burden by consumption of Zn biofortified standard flour and refined flour ranged from 10.57% to 28.38% (Table 3).

### 3.4. Bioavailability of Other Micronutrients in Wheat Flours

**Figure 4:**
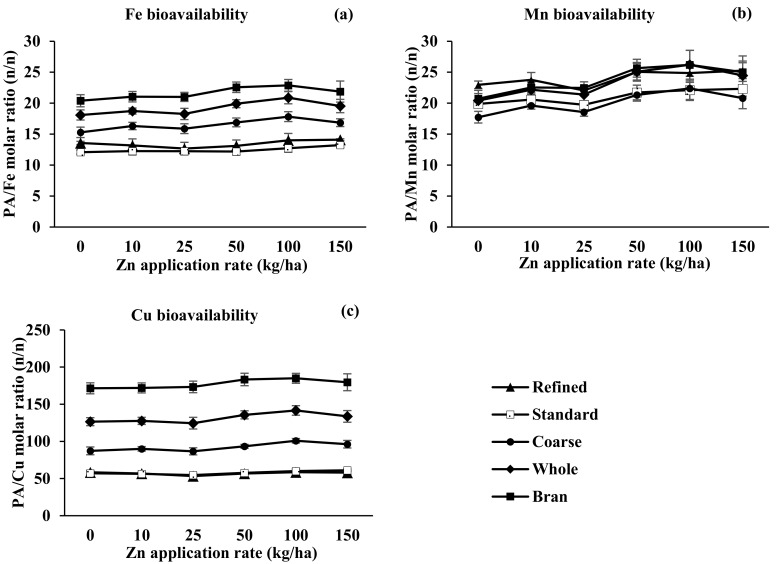
Bioavailability of Fe (**a**), Mn (**b**), and Cu (**c**) in wheat whole flour, coarse flour, standard flour, refined flour, and bran as affected by the rate of Zn fertilization. Values are means (±SE) of two cropping years (*n* = 8). PA: phytic acid.

Zinc biofortification did not significantly affect the concentrations (Table 4) and bioavailability of Fe, Mn, or Cu in the four flours or bran as indicated by the PA:Fe, PA:Mn, and PA:Cu molar ratios (Figure 4). Like Zn bioavailability, the bioavailability of Fe and Cu were higher in standard flour and refined flour than in whole flour and coarse flour. Mn bioavailability, however, did not significantly differ among the flours and bran (Figure 4).

## 4. Discussion

Zinc biofortification significantly increased Zn concentrations in grain and in all of the milling fractions (Table 2 and Figure 1). The target Zn concentration in wheat grain required to prevent Zn deficiency in humans was estimated to be 45 mg/kg [38,39]. In the current study, this target concentration was generally achieved by application of 25 kg of ZnSO_4_·7H_2_O/ha (Figure 1). The Zn concentrations were lower in standard and refined flour than in whole or coarse flour. This was mainly due to the higher Zn concentration in brans than in other fractions (Table 2). The current results were consistent with a previous finding that Zn occurs largely in the aleurone layer, in the lateral and dorsal parts of the grain [40]. The large difference in Zn concentration between bran and flour might be inherent to these fractions. That increasing Zn application rates did not increase the percentage of Zn in standard flour or bran relative to the total quantity of Zn in the wheat grain (Figure 2) indicates that the Zn application simultaneously increased the Zn concentration in all fractions of wheat grain.

During milling, the wheat grain was separated layer-by-layer, and the layers were separated into coarse bran, fine bran, and flour. These layers, however, do not precisely coincide with the layers of aleurone, endosperm, and embryo. Though there was a limitation for Zn transferred from crease, the Zn concentration in the flour increased as the Zn concentration in the grain increased. Zhang et al. [41] also reported a linear, positive correlation between Zn concentrations in wheat grain and flour. These results indicate that the higher Zn concentrations in flour and in grain can be synergistically achieved by Zn fertilization.

Although indigestible for humans and many animals, the PA and its metabolites in seeds and grains are important to the plant. PA functions as a phosphorus and energy store and as a source of cations and myoinositol (a cell wall precursor). The only undesirable property of PA is that it limits the availability of micronutrients [42]. Erdal et al. [43] found that Zn biofortification reduced PA concentrations in the seeds of 20 wheat cultivars growing in a severely Zn-deficient soil. These decreases in PA were explained by a dilution effect that occurred as a consequence of increases in grain yield in response to Zn fertilization. A needless Zn application to Zn-sufficient soils could also decrease PA concentrations in wheat grain [44]. Zhang et al. [45] reported, however, that ZnSO_4_·7H_2_O applied to soil at 50 kg/ha did not significantly affect PA concentrations in wheat products. Consistent with Zhang et al. [45], the current results indicated that the PA concentration in grain and different fractions of grain remained unchanged in response to Zn biofortification (Table 2). The DTPA-Zn of the soil in this study was 0.45 mg/kg, which indicates that the soil was not extremely Zn deficient. Because Zn biofortification increased the yield by only 6–10%, PA concentrations in grain were not substantially reduced by a dilution effect. It seems reasonable to expect that the effect of Zn fertilizers on PA concentrations in wheat grain will depend on both the levels of Zn in the soil and the Zn fertilization rate. It is also possible that wheat plants are able to regulate the PA concentration in grain under Zn stress [44].

The chemical forms of Zn and its bioavailability in food influence the amount of Zn absorbed through the gastrointestinal tract and into the bloodstream [20]. For human nutrition, Zn bioavailability in grain and flour is more important than Zn concentration. High levels of PA in food are thought to reduce the bioavailability of Zn, Fe, and other minerals [46]. Therefore, the PA:Zn molar ratio has been used to evaluate the bioavailability of Zn in food [19]. A trivariate mathematical model of Zn absorption as a function of dietary Zn, which was recently developed and tested [21], accounted for 80% of the variability in the quantity of Zn absorbed [33]. The current study showed that the estimated Zn bioavailability in grain and flour significantly increased with the rate of Zn fertilization (Figure 3), which is consistent with a previous study [47]. A daily net intestinal absorption of about 3 mg of Zn from 300 g of wheat flour consumed is required for humanhealth [14,48]. In the present study, this requirement was met only by the biofortified standard flour and refined flour (Figure 3). This is because the biofortified standard flour and refined flour contained relatively high Zn concentrations and relatively low PA concentrations. Compared with intensively processed cereals, whole grain flour and coarse flour have been traditionally thought to provide better mineral nutrition because of the higher nutrient concentration in the bran [49].

The Zn levels in the blood plasma, liver, and tibia of rats fed for 3 weeks on diets based on white wheat flour or whole flour were not significantly different, indicating that the whole flour did not reduce Zn absorption or bioavailability [50]. According to Hussain et al. [47], Zn bioavailability was higher in whole grain flour than in the other kinds of flour. In the current study, however, the estimated Zn bioavailability was lower in whole grain and coarse flour than in standard and refined flour because of the extremely high PA concentrations in the bran. The Zn concentrations were 9.5-fold higher and the PA concentrations were 15.6-fold higher in bran than in standard flour (Table 2). Thus, the whole grain flour and coarse flour, which contain more parts of the bran, had lower Zn bioavailability than standard or refined flour. This is consistent with Ryan et al. [51], who indicated that the PA:Zn and Ca × PA:Zn molar ratios were lower for flour than for whole grain. The reasons of these different findings may include differences in yield, experimental conditions, and variety. For example, the PA and Zn concentrations in wheat grain and its different fractions differ substantially among varieties [43,49].

DALYs are commonly used by the World Bank, the World Health Organization (WHO), and the HarvestPlus program [37] to assess the health burden of Zn deficiency and the health impact of biofortified wheat [23]. However, the health impact of biofortified wheat was overestimated if available Zn intake is not considered. The current study used available Zn intake as estimated by “the trivariate model” to calculate the health impact (DALYs saved) of biofortified flour and its reduction of the current health burden (Table 3). The health impacts are smaller than previously reported [23], even with the optimistic scenario of a 60% coverage rate, but might be more realistic. Still, the biofortified standard and refined flour (obtained by applying >50 kg of ZnSO_4_·7H_2_O/ha) could reduce the current healthy burden (DALYs) of Zn deficiency by more than 20%. Among all the four biofortified flours, the effects of Zn biofortification on the estimated Zn bioavailability and on human health impact (DALYs saved) were greatest for standard flour (Table 3). This was because the more available Zn intake increased with biofortification of standard and refined flour even though the total Zn concentration was lower in standard and refined flour than in whole or coarse flour. Overall, these results indicate that, like the genetic biofortification of wheat [14], the agronomic biofortification of wheat with Zn will mitigate the health burden caused by Zn deficiency among infants and children.

Unlike other studies [25,27], the present study failed to detect antagonism or synergism between Zn biofortification and the Fe, Cu, and Mn contents of wheat grain (Table 4). The soil DTPA-Fe, DTPA-Cu, and DTPA-Mn concentrations in the current study were 5.52, 0.82, and 5.30 mg/kg, respectively, all of which are higher than the critical values, indicating that the soil was not deficient in these micronutrients. The estimated bioavailability of Fe, Mn, and Cu (as indicated by the PA:Fe, PA:Mn, and PA:Cu molar ratios) was unaffected by Zn biofortification (Figure 4). Like Zn bioavailability, Fe and Cu bioavailability were higher in standard and refined flour than in whole and coarse flour. The Mn bioavailability in the four flours were not significantly different and it might be due to the similar distribution of Mn and PA in grain.

## 5. Conclusions

In the current study, Zn biofortification increased both the concentration and bioavailability of Zn in wheat grain and flours. Although Zn concentrations in wheat whole flour and coarse flour were high, Zn bioavailability and the calculated health impact (DALYs saved or reduction in the current health burden) were lower in wheat whole flour and coarse flour than in standard flour and refined flour because of the extremely high concentrations of PA in the bran. In addition, Zn biofortification did not influence the bioavailability of Fe, Mn, and Cu. The results indicate that Zn biofortification of wheat by the application of a Zn fertilizer can substantially increase the Zn bioavailability and health impact of wheat flour.

## Figures and Tables

**Table 1:** The weights and percentages of milling fractions (B1, B2, B3, R1, R2, R3, coarse bran, and fine bran) and flours in wheat grain.

	B1	B2	B3	R1	R2	R3	Coarse Bran	Fine Bran	Coarse Flour ^b^	Standard Flour ^c^	Refined Flour ^d^	Bran ^e^
Weight (g) ^a^												
2014	117.1	71.9	24.1	392.1	157.1	36.6	129.8	118.0	916.9	798.9	509.2	247.8
2015	124.4	81.1	24.6	449.8	216.6	49.9	186.3	126.7	1073.2	946.5	574.2	313.0
Percentage in grain (%)												
2014	11.2	6.9	2.3	37.5	15.0	3.5	12.4	11.3	87.6	76.3	48.7	23.7
2015	9.9	6.4	2.0	35.7	17.2	4.0	14.8	10.1	85.2	75.1	45.6	24.9
Average	10.5	6.6	2.1	36.6	16.1	3.7	13.6	10.7	86.4	75.7	47.1	24.3

^a^ The Bühler laboratory experimental mill (MLU-202) required about a 1-kg sample to produce different milling fractions. The average weight of total wheat grain was 1046.8 and 1259.5 g in 2014 and 2015, respectively; ^b^ A mixture of B1, B2, B3, R1, R2, R3, and fine bran fractions; ^c^ A mixture of B1, B2, B3, R1, R2, and R3 fractions; ^d^ A mixture of B1 and R1 fractions; ^e^ A mixture of coarse bran and fine bran fractions.

**Table 2:** Zinc and phytic acid concentrations in milling fractions (B1, B2, B3, R1, R2, R3, coarse bran, and fine bran) of winter wheat as affected by the rate of Zn biofortification via fertilizer application.

ZnSO_4_·7H_2_O Rate (kg/ha)	B1		B2		B3		R1		R2		R3		Coarse Bran		Fine Bran	
	2014	2015	2014	2015	2014	2015	2014	2015	2014	2015	2014	2015	2014	2015	2014	2015
Zn concentration (mg/kg)																
0	11.4 d	10.8 d	13.2 d	5.7 e	22.2 d	18.9 c	10.3 d	9.2 d	12.4 d	12.0 d	24.6 c	26.7 c	106 e	98.3 e	114 d	105 e
10	13.1 cd	12.4 cd	15.1 c	7.6 de	25.9 c	25.5 b	12.0 c	11.5 c	15.2 c	13.7 cd	32.2 b	31.4 b	128 d	120 d	140 c	123 de
25	13.9 c	13.5 cd	16.1 c	9.4 cd	26.5 bc	24.4 b	12.9 c	12.9 bc	16.4 bc	14.9 bc	37.9 b	32.3 b	142 cd	137 cd	163 b	138 cd
50	14.2 bc	14.9 bc	16.4 bc	10.7 bc	28.1 abc	27.5 b	13.6 bc	13.5 b	18.5 b	15.7 b	46.4 a	34.3 b	149 bc	152 bc	166 b	154 bc
100	16.1 ab	17.0 ab	17.8 ab	11.6 b	28.6 ab	27.5 b	14.9 ab	14.2 ab	21.1 a	16.1 b	49.7 a	34.2 b	165 ab	163 ab	185 a	159 b
150	17.4 a	19.1 a	18.4 a	13.8 a	29.6 a	31.5 a	15.4 a	15.5 a	21.2 a	18.2 a	45.8 a	41.9 a	171 a	177 a	193 a	186 a
Phytic acid concentration (g/kg)																
0	1.68 a	1.63 a	2.11 b	1.76 bc	1.85 a	1.38 a	1.62 a	1.20 a	1.94 b	1.47 a	2.40 a	3.06 a	28.1 a	26.2 a	22.4 b	15.6 a
10	1.91 a	1.60 a	2.21 ab	1.77 bc	1.69 a	1.53 a	1.56 a	1.05 a	2.09 b	1.49 a	3.07 a	2.86 a	26.5 a	26.0 a	25.3 ab	17.3 a
25	1.80 a	1.58 a	2.16 b	1.67 c	1.82 a	1.47 a	1.57 a	1.05 a	2.05 b	1.39 a	3.28 a	3.46 a	25.6 a	27.9 a	22.0 b	19.3 a
50	1.83 a	1.63 a	2.18 ab	1.70 c	1.62 a	1.52 a	1.51 a	1.19 a	2.45 a	1.39 a	3.30 a	2.85 a	28.1 a	30.1 a	25.0 ab	18.5 a
100	1.91 a	1.66 a	2.20 ab	1.91 ab	1.90 a	1.64 a	1.49 a	1.14 a	2.55 a	1.32 a	3.70 a	3.25 a	29.1 a	28.7 a	29.0 a	19.3 a
150	2.03 a	1.70 a	2.40 a	2.06 a	1.75 a	1.51 a	1.60 a	1.14 a	2.62 a	1.46 a	3.72 a	3.07 a	28.8 a	29.0 a	27.1 ab	22.1 a

Values are means of four replications for each of two growing seasons (2014 and 2015). Means followed by different letters are significantly different at *p* < 0.05.

**Table 3:** Health impact of Zn biofortified flours in China.

Parameter	Population	Fertilization Rate (kg of ZnSO_4_·7H_2_O/ha)					
		0	10	25	50	100	150
Whole flour							
Daily Zn intake (mg per day)	Infants	4.90	4.96	5.01	5.02	5.04	5.08
	Children	6.00	6.12	6.21	6.24	6.28	6.35
% of recommended nutrition intake (RNI)	Infants	71.00	71.87	72.57	72.73	73.06	73.56
	Children	71.50	76.47	77.68	77.96	78.52	79.39
Health impact (DALYs saved)	Infants	-	35,451	63,343	69,793	82,377	101,367
	Children	-	196,418	345,503	379,258	444,317	540,416
% reduction in the current health burden ^a^		-	6.58	11.60	12.74	14.94	18.21
Coarse flour							
Daily Zn intake (mg per day)	Infants	4.90	4.96	5.01	5.05	5.04	5.08
	Children	6.00	6.11	6.22	6.29	6.29	6.36
% of recommended nutrition intake (RNI)	Infants	71.00	71.81	72.63	73.15	73.10	73.63
	Children	71.50	76.38	77.79	78.69	78.60	79.51
Health impact (DALYs saved)	Infants	-	33,188	65,982	86,042	84,009	104,023
	Children	-	184,104	359,348	463,061	452,676	553,656
% reduction in the current health burden		-	6.17	12.07	15.58	15.23	18.66
Standard flour							
Daily Zn intake (mg per day)	Infants	4.90	5.00	5.06	5.10	5.13	5.16
	Children	6.00	6.19	6.32	6.41	6.45	6.52
% of recommended nutrition intake (RNI)	Infants	71.00	72.42	73.32	73.97	74.29	74.79
	Children	71.50	77.42	78.98	80.11	80.65	81.51
Health impact (DALYs saved)	Infants	-	57,566	92,482	116,774	128,089	145,901
	Children	-	315,033	495,770	616,480	671,209	755,339
% reduction in the current health burden		-	10.57	16.69	20.81	22.68	25.57
Refined flour							
Daily Zn intake (mg per day)	Infants	4.90	5.02	5.08	5.10	5.17	5.20
	Children	6.00	6.24	6.37	6.40	6.54	6.59
% of recommended nutrition intake (RNI)	Infants	71.00	72.75	73.62	73.91	74.93	75.36
	Children	71.50	78.00	79.63	80.00	81.75	82.38
Health impact (DALYs saved)	Infants	-	70,694	103,797	114,507	150,744	165,688
	Children	-	383,952	565,899	605,399	777,772	834,668
% reduction in the current health burden		-	12.90	19.00	20.43	26.35	28.38

^a^ % reduction in the current health burden = Health impact (DALYs saved) of Zn biofortified flour/health burden without biofortification × 100. In this equation, health burden is equivalent to DALYs.

**Table 4:** Fe, Mn, and Cu concentrations in whole flour, coarse flour, standard flour, refined flour, and bran as affected by the Zn fertilization rate. Values are the means of four replications for each of two growing seasons (2014 and 2015). Means in a column followed by different letters are significantly different at *p* < 0.05.

ZnSO_4_·7H_2_O Application Rate (kg/ha)	Whole Flour		Coarse Flour		Standard Flour		Refined Flour		Bran	
	2014	2015	2014	2015	2014	2015	2014	2015	2014	2015
Fe concentration (mg/kg)										
0	39.6 a	34.5 a	27.3 a	22.0 a	12.7 a	10.3 a	10.5 a	8.0 a	106.3 a	95.3 a
10	38.3 a	34.0 a	26.0 a	20.8 a	12.0 a	10.2 a	9.3 a	9.2 a	104.0 a	93.0 a
25	40.0 a	34.4 a	26.6 a	21.2 a	12.8 a	9.5 a	10.8 a	8.4 a	106.6 a	95.0 a
50	37.3 a	34.5 a	26.2 a	20.8 a	12.6 a	10.3 a	9.5 a	9.3 a	101.1 a	93.0 a
100	38.2 a	32.4 a	27.2 a	19.9 a	13.0 a	9.3 a	9.3 a	8.1 a	104.1 a	88.5 a
150	39.0 a	33.1 a	28.0 a	20.8 a	13.1 a	9.3 a	10.0 a	8.5 a	105.3 a	92.9 a
Mn concentration (mg/kg)										
0	37.0 a	28.9 a	24.0 a	17.9 a	7.3 a	5.9 a	5.8 a	4.9 a	110.2 a	86.9 a
10	35.8 ab	25.3 ab	22.9 a	15.2 ab	7.4 a	5.6 a	5.6 a	4.2 a	109.5 a	75.1 ab
25	37.6 a	26.4 ab	24.4 a	16.3 ab	7.8 a	5.7 a	6.1 a	4.5 a	111.5 a	77.9 ab
50	34.0 ab	23.7 b	23.4 a	14.1 b	7.7 a	5.1 a	5.7 a	4.0 a	104.7 a	69.6 b
100	33.2 b	23.7 b	23.1 a	14.7 b	7.6 a	5.1 a	5.5 a	4.1 a	102.5 a	69.8 b
150	33.5 b	24.7 ab	23.5 a	16.1 ab	7.4 a	5.6 a	5.5 a	4.3 a	102.5 a	73.4 ab
Cu concentration (mg/kg)										
0	6.9 a	5.5 a	5.8 a	4.2 a	3.3 a	2.3 a	2.9 a	2.0 a	14.5 a	13.0 a
10	6.7 a	5.4 a	5.6 a	4.0 a	3.3 a	2.3 a	2.8 a	2.0 a	14.6 a	12.9 a
25	6.9 a	5.6 a	5.8 a	4.3 a	3.3 a	2.4 a	3.0 a	2.1 a	15.2 a	13.1 a
50	6.5 a	5.6 a	5.5 a	4.2 a	3.2 a	2.4 a	2.8 a	2.1 a	14.3 a	12.9 a
100	6.6 a	5.3 a	5.6 a	4.0 a	3.3 a	2.1 a	2.9 a	1.9 a	14.4 a	12.7 a
150	6.7 a	5.4 a	5.8 a	4.1 a	3.4 a	2.2 a	3.1 a	1.9 a	14.4 a	13.0 a
